# Enhanced Induction Heating and Self-Healing Properties of Steel Slag Powder Based Asphalt and Asphalt Mixture under Microwave Irradiation

**DOI:** 10.3390/ma16093312

**Published:** 2023-04-23

**Authors:** Hao Xu, Mingzhi Sun, Guobao Luo

**Affiliations:** 1 School of Civil Engineering, Chongqing Jiaotong University, Chongqing 400074, China; 2 Research Institute of Highway Ministry of Transport, Beijing 100088, China; gb.luo@rioh.cn

**Keywords:** road engineering, asphalt self-healing, microwave heating, steel slag powder

## Abstract

This paper aims to study the application feasibility of steel slag powder (SSP) in replacing limestone powder (LP) to enhance the heat release and self-healing properties of asphalt and an asphalt mixture. First, the microwave-heating characteristics of SSP and LP asphalt mortar were analyzed, and the differences in the microstructure and chemical composition between SSP and LP were compared. Secondly, through the DSR frequency sweep test, the optimal healing temperature of the two asphalt mortars was determined. Finally, asphalt mixtures with different SSP contents were prepared by replacing part of LP in the mixture in a gradation with SSP. Under microwave radiation, the temperature distribution of the mixture was explored, and the self-healing properties and factors affecting the healing were analyzed. Results demonstrated that there are metal oxides with high electromagnetic parameters such as Fe_2_O_3_ and CaO in SSP, therefore, asphalt and a mixture containing SSP were seen to have excellent microwave absorption capacity. The healing temperature of the two kinds of asphalt mortar was between ~50 °C and 60 °C. Under microwave radiation, the temperature of the asphalt mixture increased with the increase in SSP content, and the temperature difference decreased with the increase in SSP content. Asphalt mixtures with an LP content of 30%, 40%, 50%, 60%, and 70% replaced by SSP increased the healing index by 8.7%, 17.3%, 22.1%, 26.9%, and 27.7% compared with conventional asphalt mixtures. Temperature is the most important factor affecting the healing behavior of the asphalt mixture. With the increase in the damage times of the asphalt mixture, the overall healing index of the asphalt mixture showed a downward trend. However, the healing index of an asphalt mixture containing SSP can still be maintained at more than 50% after repeated mechanical damage.

## 1. Introduction

The main failure form of asphalt pavement is a crack and it has a significant negative influence on pavement performance. How to slow down the development of cracks and prolong the service life of the pavement is a concern of many researchers. Asphalt has rheological properties and viscoelastic properties. After being damaged by external forces, micro-cracks and micro-pores inside asphalt can be closed under certain conditions so that asphalt performance can recover to a certain extent, which is called the self-healing property of bitumen [1,2,3,4,5]. Road workers can take advantage of this property to pre-conserve the asphalt pavement before macroscopic damage occurs to extend the service life of the pavement.

Thermal induction self-healing technology heats the asphalt mixture through electromagnetic induction, microwave, infrared and other methods to enhance the fluidity of asphalt, thus inducing the self-healing characteristics of the asphalt, and making the asphalt cracks heal, restoring part of the strength of the asphalt mixture [6,7,8,9]. Microwaves have the advantages of fast heating speeds, heating without hysteresis effects, and creating uniform temperature fields, so they have been studied by the majority of researchers. Common microwave-absorbing materials in road engineering are metal waste, metal oxide, steel fiber, carbon fiber, etc. [10,11,12].

According to Juan Gallego et al. [13], compared with electromagnetic induction and infrared, microwave thermal induction technology is more efficient in healing asphalt road damage. Norambuena-Contrera et al. [14] found that the healing level of the asphalt mixture decreased with each healing cycle under repeated healing tests with a steel velvet fiber asphalt mixture. Zhu Xingyi et al. [15,16] conducted a microwave heating self-healing test on an open-grade friction course (OGFC) asphalt mixture with 5% ferrite and found that ferrite can significantly delay secondary crack development and reduce the crack growth rate. At the same time, the healing effect was proportional to the healing time, but the recovery time had a smaller effect on the self-healing than the heating time. Gonzalez et al. [17] found that adding metal fibers to the asphalt mixture could promote its microwave self-healing. Zhu Hongzhou et al. [18,19] studied the factors influencing the self-healing performance of an asphalt mixture, and their results showed that microwave intensity had the most significant effect, followed by the heating time. The self-healing ability was directly proportional to microwave intensity and heating time, and the damage degree had an adverse effect on the self-healing performance. By studying the temperature field of an asphalt mixture, Sun Yihan [20] found that the temperature distribution of an asphalt mixture under microwave irradiation was significantly more uniform than that under electromagnetic induction heating, and the effective depth of microwave heating technology on the asphalt mixture was about 10 cm. All the above studies on thermal induction techniques were carried out on a single mixture layer without establishing a connection with the asphalt or asphalt mortar layer. Wang, Fu et al. [21] studied, through the heating rate test, semi-circular bending test (SCB) test, and fatigue test of an asphalt mixture, its heating characteristics, flexural strength, fatigue resistance, and self-healing performance. The results showed that, after microwave heating, the flexural strength and fatigue resistance of an asphalt mixture with basalt and limestone aggregates can recover at least 65% and 23%, respectively. Wang Qiang et al. [22] prepared an asphalt mixture by replacing part of the limestone with steel slag and studied its self-healing properties under microwave heating. The results showed that the steel slag asphalt mixture self-healing index fracture energy healing rate was noticeably higher than that of the conventional mixture.

Steel slag is a by-product produced in the process of steelmaking, and its output is about 8–15% of the output of crude steel. China produces more than 100 million tons of steel slag every year, but the effective utilization rate is less than 30%, and the accumulated steel slag is more than 1 billion tons. The long-term accumulation of steel slag is prone to wind and rain erosion, leading to steel slag pulverization and metal ion leaching, which causes serious pollution to air, water, and soil resources [23,24,25]. Using steel slag in road construction is an effective means of solid waste treatment [26].

Steel slag, which mainly consists of oxides of various elements and impurities of metal raw materials, is a good microwave-absorbing material [27,28,29]. In many previous studies, the self-healing properties of a single layer (asphalt or mixture) have been analyzed, though a comprehensive and systematic study is lacking. In this paper, the feasibility of the microwave-induced self-healing technology of steel slag powder was verified at two levels of binder and mixture. Firstly, the microwave heating test of asphalt mortar containing steel slag powder (SSP) and limestone powder (LP) was carried out to analyze the microwave heating effect of SSP and LP. The optimum self-healing temperature of the two kinds of asphalt mortar was determined by a frequency scanning test through a dynamic shear rheological test. Secondly, the asphaltic mixture was prepared with SSP instead of LP in a mixture gradation, and a self-healing test was carried out to determine the optimal replacement amount of SSP. Finally, the influence factors of microwave self-healing technology were analyzed. The purpose of this paper is to provide a reference for the realization of microwave self-healing technology of asphalt pavement in road engineering.

## 2. Materials and Methods

### 2.1. Raw Materials

The asphalt used in this research is Sinopec Dong hai 70 # matrix asphalt. Limestone aggregate and limestone powder (LP) were obtained from Chong Jiao Renewable Resources Development Co., Ltd. Chongqing, China. Steel slag powder (SSP) was obtained from Jingye Steel Factory, Hebei, China. The basic properties of the raw materials are concluded in Table 1, Table 2 and Table 3.

### 2.2. Experimental Methods

#### 2.2.1. Preparation of Asphalt Mortars

Asphalt mortars with four filler-bitumen ratios were designed, and the mass ratio of filler to asphalt was 0, 0.6, 0.9, and 1.2. First, the mass of the filler was weighed. According to Table 2, the density of SSP is greater than that of LP; therefore, the equivalent substitution method was used, LP was replaced by equal volume SSP, the mass of the designed powder-binder ratio LP was determined, and then the SSP mass was calculated according to Equation (1). The filler was added to the asphalt that was kept in an oil bath at 150 °C for 4 h. The mixture of filler and asphalt was stirred with a mixer at a speed of 1500 rad/min for 3 min and asphalt mortar preparation was completed.

(1) $$m_{s}=m_{f}*\frac{\rho_{s}}{\rho_{f}}$$

In Equation (1), $m_{s}$ represents the mass of SSP, $m_{f}$ represents the mass of LP, $\rho_{s}$ represents the density of SSP, and $\rho_{f}$ represents the density of LP.

#### 2.2.2. Preparation of Asphalt Mixture Specimen

**Figure 1:**
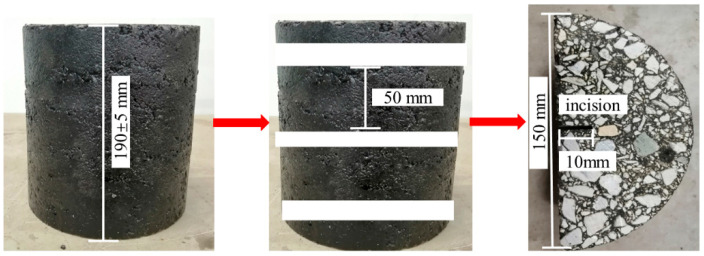
The cutting process of the specimen.

In this study, part of LP in the gradation was replaced by equal volume SSP, and cylindrical asphalt mixture specimens with different substitution amounts of SSP (0%, 30%, 40%, 50%, 60%, and 70%) were prepared following the gyratory compactor method with the test parameters set as follows: compressive stress = 600 KPa, offset angle = 1.16°, and rotation rate = 30 r/min; height control (170 mm) was selected as the compaction control mode. As described in Table 4, AC-13 type mixture gradation was adopted, and the optimum oil-aggregate ratio of the asphalt mixture was 4.7%. The air void content of all types of the mixture was 4 ± 0.5%. Cylindrical specimens needed to be cut into semicircular specimens. The diameter of the semicircular specimen was 150 mm and the thickness was 50 mm. In order to avoid permanent deformations and to obtain a brittle fracture during the semi-circular bending test, a notch with a width of 2 mm and a length of 10 mm was cut in the center of the bottom of the specimen. The preparation process of the test piece is shown in Figure 1.

#### 2.2.3. Microwave Heating of Asphalt Mortar and Mixture

Microwave heating test of asphalt mortar: Asphalt mortar samples with a diameter of 2 cm and a thickness of 1 cm with different filler–bitumen ratios were prepared, and the samples were placed in groups of four on a glass plate, with each specimen being 3 cm apart. The samples of different asphalt mortars were divided into two groups. The first group consisted of SSP asphalt mortar with four filler-bitumen ratios (0, 0.6, 0.9, and 1.2). The second group consisted of LP asphalt mortar with the same four filler–bitumen ratios. Pure asphalt was the controlled asphalt mortar and was recognized as having a filler–bitumen ratio of 0. Each group was heated four times continuously (20 s each time) through microwave heating equipment. Meanwhile, an infrared camera was used to record the temperature distribution after each heating time.

Microwave heating test of asphalt mixture: In order to research the effect of SSP on the overall heating performance of the mixture, the stone and asphalt mixtures with different SSP substitution amounts were heated by continuous microwave for 80 s, and the temperature distribution was acquired by the use of an infrared camera. An NN-GT353M microwave oven produced by Panasonic, Japan was used as a microwave heating device, and the heating power and frequency of the generated microwave were 800 W and 2.45 GHz, respectively. The infrared thermal imager was an AvioR300 from Japan, with a temperature range of −40 °C to 500 °C and a temperature resolution of 0.03 °C.

In order to explain the mechanism of SSP microwave absorption, the chemical composition of SSP and LP were tested with an X-ray fluorescence spectrometer (XRF), the microstructure of SSP and LP was observed by SEM, the mineral composition of the SSP was tested by X-ray diffraction (XRD).

#### 2.2.4. Healing Temperature of Asphalt Mortar

García et al. found that asphalt will present a near-Newtonian fluid state under certain temperature conditions and that that temperature range is between 30 °C and 70 °C [30,31]. To determine the optimal healing temperatures of asphalt mortar when near-Newtonian behavior occurs Dynamic Shear Rheometer (DSR) was used to measure the rheological properties of asphalt mortar. SSP asphalt mortar and FP asphalt mortar with different filler–bitumen ratios were used as the experiment and the test parameters were set as follows: test temperature range = 40 °C~80 °C, temperature interval = 10 °C, and scanning frequency 0.1~10 Hz.

#### 2.2.5. Healing Properties of Asphalt Mixture

**Figure 2:**
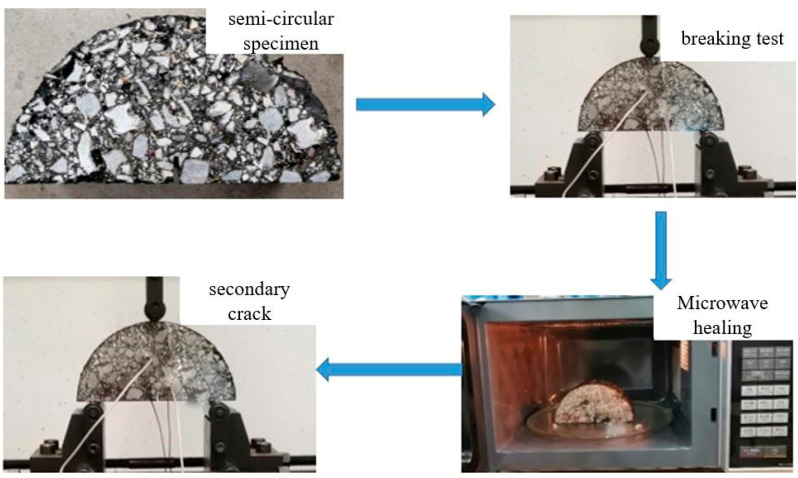
The self-healing experimental process of the asphalt mixture.

The “fracture-healing-refracture” experiment was used to evaluate the self-healing properties of semicircular specimens with different SSP substitutions, The fracture test equipment was the Mester electronic universal testing machine; in order to eliminate the influence of asphalt creep on its self-healing properties the test temperature was set to −10 °C and the loading rate was 0.50 mm/min. After the specimen fractured, the spliced fractured specimen was placed in a microwave oven for heating and healing. The healing time was 3 min, and the temperature was measured every minute with an infrared camera during the healing process. For asphalt specimens with a high SSP substitution, when the temperature of the specimen exceeded the predetermined healing temperature, the heating was stopped, and the heating continued after the temperature decreased. After microwave healing was completed, the specimen was cured at room temperature for 2 h to cool, and to ensure that the fracture surface was in contact, two rubber bands (elastic modulus of about 0.0784 MPa) were used to fix the specimen. The tensile force of each rubber band was about 10 N. After the healing was completed, the next fracture test was performed. The experimental process is shown in Figure 2. The self-healing index was calculated according to Equation (2).

(2) $$HI=\frac{F_{1}}{F_{0}}*100\%$$

In Equation (2), *HI* represents the healing index of the asphalt mixture, $F_{1}$ represents the maximum breaking strength of the first fracture, and $F_{0}$ represents the maximum breaking strength of the second fracture.

#### 2.2.6. Self-Healing Factors of Asphalt Mixture

1. Conventional influencing factors:

Orthogonal experiments were designed to analyze the influence of various factors on the healing properties of asphalt. In the process of microwave heating asphalt mixture healing, the specimen healing temperature, healing time, and damage degree are considered to be the main factors that affect the healing of the asphalt mixture. The changes in the three factors are controlled by the microwave heating time, normal temperature curing time, and vertical displacement of the indenter of the universal testing machine. Among them, the degree of injury refers to the damage degree of the asphalt mixture semi-circle specimen under the action of external force. In the test, the larger the vertical displacement of the universal testing machine’s indenter, the more serious the damage degree of the specimen influencing factors and levels as shown in Table 5.

2. Effects of multiple mechanical injuries on self-healing properties.

During the service of the road, the asphalt road was repeatedly run over by vehicles and cracks appeared. Therefore, several “fracture-healing-refracture” experiments were designed to evaluate the self-healing performance changes of asphalt mixtures during long-term service.

## 3. Results and Discussion

### 3.1. Microwave Heating Properties of Asphalt Mortar

**Figure 3:**
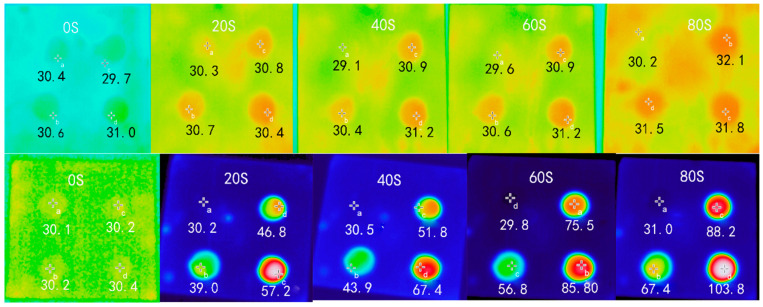
Infrared image of asphalt mortar (the (**top**) is LP asphalt mortar, the (**bottom**) is SSP asphalt mortar).

**Figure 4:**
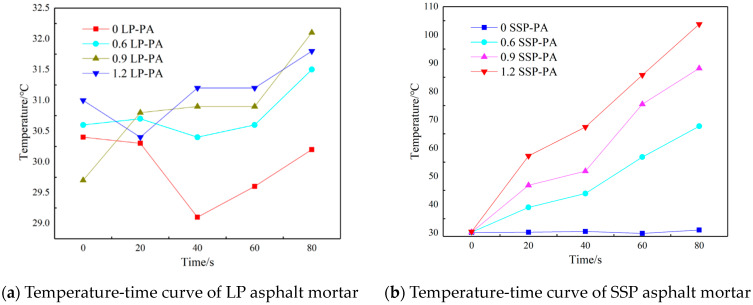
The heating curve of asphalt mortars under microwave irradiation.

Infrared images of different asphalt mortars under microwave action are shown in Figure 3. Asphalt mortar with four filler-bitumen ratios of 0, 0.6, 0.9, and 1.2 are represented by the top left, bottom left, top right, and bottom right circular areas in the infrared image. The center temperature point of the circular area was marked. In order to analyze the heating area more accurately and intuitively, the heating curves of the asphalt mortar with different ratios of limestone powder-pure asphalt (LP-PA) and steel slag powder-pure asphalt (SSP-PA) are shown in Figure 4.

Several trends are obvious from Figure 3 and Figure 4; with the increase in microwave heating time, the temperature of the LF mortar slurry almost did not change. On the contrary, the temperature of the SSP asphalt mortar increased with increasing microwave heating. The SSP asphalt mortars with SSP-PA ratios of 0.6, 0.9, and 1.2 increased from 30.1 °C to 67.4 °C, 88.2 °C, and 103.8 °C, respectively, and the average increasing rates were 0.466 °C/S, 0.726 °C/S, and 0.921 °C/S, respectively. The reason is that there are metal oxides with high electromagnetic parameters such as Fe_2_O_3_ and CaO in SSP, which can absorb the microwave well and convert the absorbed electromagnetic energy loss into heat. However, the main component of limestone is CaCO_3_, whose electromagnetic parameters are far smaller than those of metal oxide, and its ability to absorb and convert microwaves is very weak.

**Figure 5:**
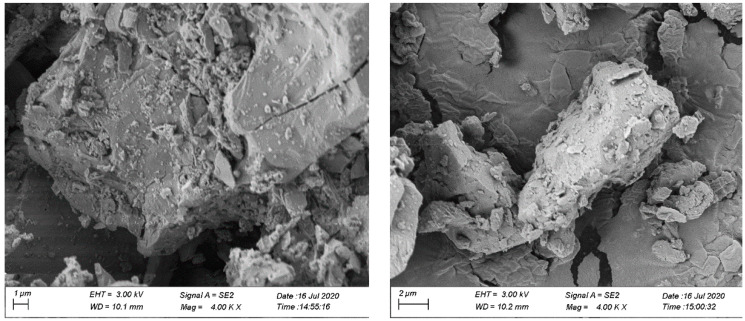
Microscopic appearance of the filler after 4000 times magnification ((**left**) SSP, (**right**) LP).

**Figure 6:**
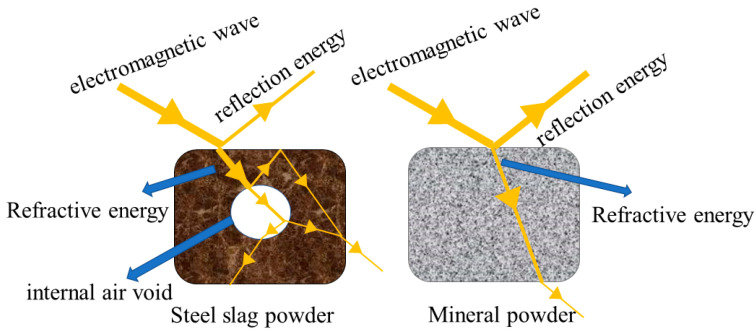
Schematic diagram of structure features.

The microstructure of SSP and limestone LP was observed by SEM. The results are shown in Figure 5, and the structural characteristics of the two fillers were drawn as shown in Figure 6.

It can be seen in Figure 5 and Figure 6 that, compared with LP, SSP has a rough surface structure and is porous. This special surface structure has an important influence on its microwave absorption ability. Microwaves can have multiple reflections and refractions in the inner part of SSP due to its porous structure. The energy of the SSP reflection microwave was less than LP and the energy reflecting the microwave was greater than in LP. Electromagnetic waves were repeatedly attenuated inside the steel slag and the consumed microwave energy was converted into heat.

**Figure 7:**
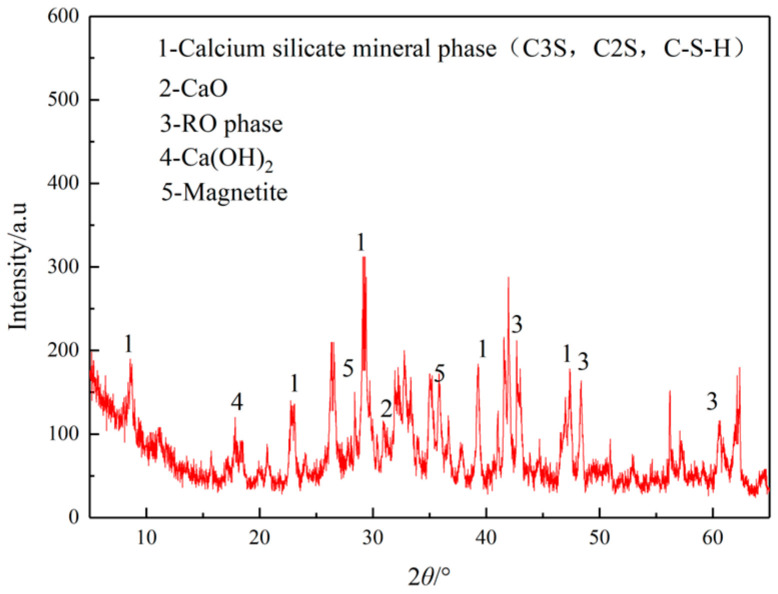
XRD test results of SSP.

Table 6 shows the XRF test results of the SSP and LP and Figure 7 shows the XRD test results of the SSP.

It can be seen from Table 6 that the main chemical compositions of the SSP were SiO_2_, Al_2_O_3_, CaO, and Fe_2_O_3_, while the main component of LP was CaCO_3_. Figure 7 shows the XRD results of the SSP. The SSP contains multiple minerals. The main mineral components of the SSP were magnetite, the RO phase, dicalcium silicate (C2S), and tricalcium silicate (C3S); the magnetite especially is considered as hyperactive for microwave heating. The SSP also contained a certain amount of Ca(OH)_2_ which endowed the SSP with strong alkalinity so that it could react with the anhydride in asphalt to enhance the bonding force between the SSP and asphalt.

### 3.2. Research on the Optimum Healing Temperature of Asphalt Mortar

**Figure 8:**
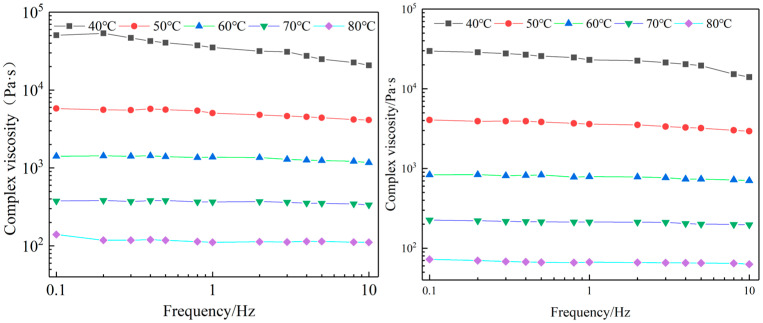
The complex viscosity frequency curve of asphalt mortar with a filler–bitumen ratio of 0.6 ((**left**): SSP asphalt mortar, (**right**): LP asphalt mortar).

Figure 8 shows the effect of frequency on the complex viscosity (n) of the asphalt mortar with a filler–bitumen ratio of 0.6 viscosity at different temperatures. In order to find out at what temperature this asphalt mortar will start flowing, the rheological data of the asphalt mortar was fitted through the following power law, Equation (3).

(3) $$\eta^{*}=m|\omega {|}^{n-1}\;$$

**Figure 9:**
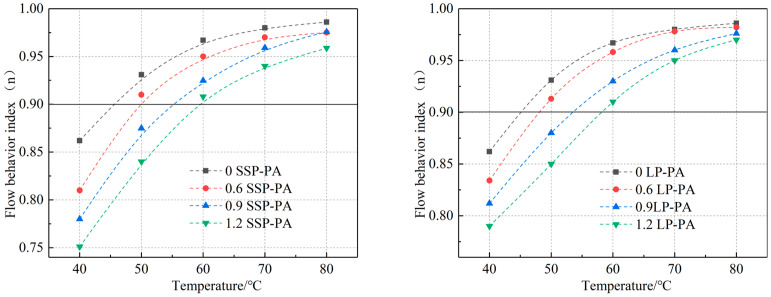
The variation curve of asphalt mortar flow behavior index n with temperature. ((**left**): SSP asphalt mortar, (**right**): LP asphalt mortar).

In Equation (3), ω represents the frequency, $\eta^{*}$ represents the complex viscosity, and *m* and n represent the fitting parameters. N is also called the flow behavior index (n). When *n* = 1, the material is in a Newtonian fluid state. When the *n* reaches 0.9, which is usually seen as a transition temperature, asphalt can act like a near-Newtonian fluid at this temperature. According to the literature [20] and [32], the temperature corresponding to *n* = 0.9 was selected as the optimum healing temperature of asphalt mortar in this research. The variation curve of asphalt mortar flow behavior index n with temperature is shown in Figure 9 and the optimum healing temperature of different types of asphalt mortar is shown in Table 7.

It can be seen from Figure 8 that the complex viscosity decreases with the increase of frequency at 40 °C and 50 °C, however, at higher temperatures (70 °C and 80 °C), the trend of the curve was relatively flat. At high temperatures, the asphalt mortar approaches or reaches a Newtonian fluid state, so the change of viscosity with frequency remains almost constant in value. At the same frequency and temperature, the composite viscosity of SSP asphalt mortar is obviously higher than that of LP asphalt mortar.

Several trends are obvious from Figure 9, for instance, the flow behavior index (n) increases with temperature. In the temperature range of 45 °C~60 °C, the flow behavior index of the two types of asphalt mortar with filler–bitumen ratios of 0, 0.6, 0.9, and 1.2 can be increased to above 0.9. At the same temperature, as the filler–bitumen ratio increases, the flow behavior index of the mortar becomes significantly lower, and a higher temperature was required to increase the flow behavior index of the mortar to 0.9. It can also be seen that the flow performance of SSP asphalt mortar is slightly worse than that of LP asphalt mortar, which is related to the nature of SSP itself and its rough surface structure. The bonding ability and adhesion between SSP and asphalt are stronger than that of LP. Table 7 shows that the optimum healing temperature of the two types of asphalt mortar increases with the increase of the filler–bitumen ratio, and the healing temperature of SSP asphalt mortars with filler–bitumen ratios of 0.6, 0.9, and 1.2 were 1.2 °C, 2.2 °C, and 2.6 °C higher than that of LF asphalt mortar, respectively, but the optimum healing temperatures of both were between 50 °C and 60 °C. The filler–bitumen ratio in the dense asphalt mixture was around 1.2. Therefore, when studying the self-healing performance of the asphalt mixture in the following chapters, it is more reasonable to use a temperature around 60 °C as the microwave-induced healing temperature of the asphalt mixture.

### 3.3. Microwave Heating Characteristics of Asphalt Mixture

**Figure 10:**
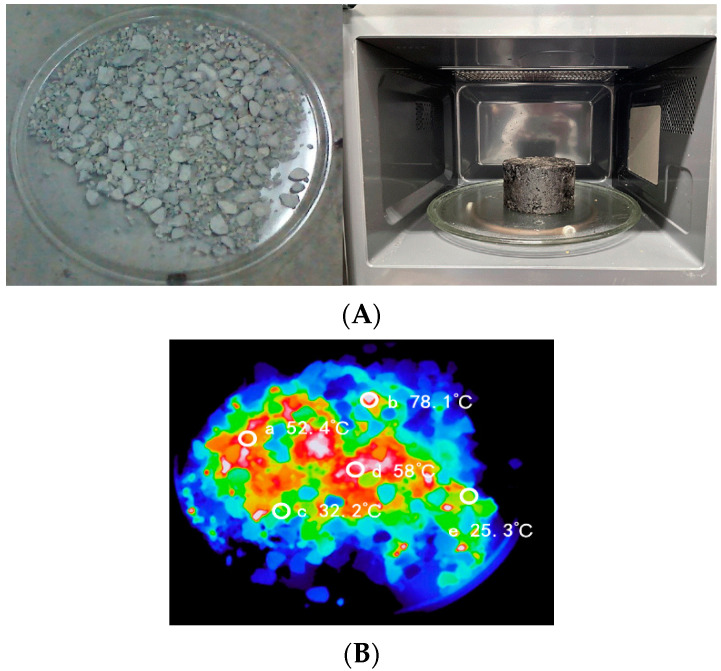

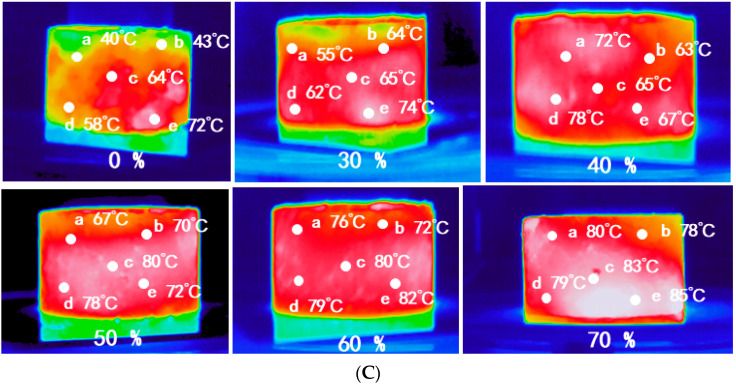
The infrared thermal images of the aggregate and asphalt mixture surface temperature and their corresponding ordinary optical images. (**A**) Ordinary optical photos of aggregate and SSP asphalt mixture; (**B**) infrared thermography of aggregate surface temperature; and (**C**) infrared thermal image of asphalt mixture with different replacements of SSP.

The asphalt mixture consisted of asphalt and aggregate. According to the above, it can be known that pure asphalt has a limited microwave absorption capacity. This section focuses on the microwave absorption capacity of the aggregate itself and the asphalt mixture with different steel slag replacement amounts. The infrared thermography of the limestone aggregate surface temperature and the surface temperature of the asphalt mixture with different replacements of SSP after microwave heating for 2 min and their ordinary optical images is shown in Figure 10.

It can be seen from Figure 10B that the highest temperature on the surface of limestone aggregates reached 78.1 °C, but the lowest temperature was only 25.3 °C, after 2 min of microwave heating. At the same time, it can be seen that there are three temperature centers, namely: 78 °C, 58 °C, and 52 °C. The temperature distribution of limestone aggregates was uneven under microwave radiation, and there was a phenomenon of temperature concentration. The reason for this phenomenon is that the particle size and shape of limestone aggregates are different, and the absorption and reflection of the microwaves are different. The particle size and shape will obviously affect its heating characteristics. At the same time, the material composition of limestone aggregates affects their ability to absorb microwaves. As the main component of limestone, CaCO_3_ has weak microwave absorption. However, there are some metal compounds (magnetite, hematite, etc.) on the surface or inside of the rock during the formation process, and the metal compounds can absorb microwaves very well, so the local temperature of the aggregate will be too high under the action of microwaves.

Several trends were obvious from Figure 10C; under the action of microwave, the temperature of the asphalt mixture increases with the increase of SSP substitution, and the temperature difference decreases continuously with the increase in substitution. The temperature differences of asphalt mixtures with SSP substitutions of 0%, 30%, 40%, 50%, 60%, and 70% were: 32 °C, 19 °C, 15 °C, 13 °C, 10 °C, and 7 °C. The overall temperature rise of the ordinary asphalt mixture specimens was achieved by transferring heat to the asphalt through heat conduction after the aggregate was heated up. Due to the temperature concentration phenomenon of the aggregate under microwave radiation, the overall temperature of the specimen was uneven. The temperature of the SSP asphalt mixture specimen has two sources, one is the aggregate temperature rise and the other is the SSP asphalt mortar temperature rise. Since the SSP asphalt mortar wraps the aggregate and the SSP has a stronger microwave absorption capacity than the aggregate, as the SSP content increases, the overall temperature of the specimen becomes more and more uniform.

### 3.4. Effect of SSP Substitution on Self-Healing Properties of Asphalt Mixture

The healing indexes of asphalt mixtures with different SSP substitutions under microwave thermal induction are shown in Table 8, and the healing temperature was controlled at about 60 °C.

It can be seen from Table 8 that the maximum fracture force of semicircular specimens of different types of asphalt mixtures increases with the increase in steel slag replacement. It shows that the rough surface structure of SSP has a stronger binding ability to asphalt than LP, and the fracture surface of the specimen was actually the fracture of asphalt mortar. At the same time, the essence of the healing of cracks in the semicircular asphalt mixture specimens is the healing of the asphalt mortar. In microwave heating healing, with the increase in the SSP content in the specimen, the asphalt mortar can reach the set healing temperature range, so that the test parts achieve the desired healing effect. However, under microwave radiation, the ordinary asphalt mixture without SSP content has a slow heating rate and uneven temperature distribution, and the overall asphalt mortar cannot reach the healing temperature well, resulting in the poor overall fluidity of the asphalt mortar on the fracture surface of the specimen.

The healing indexes of asphalt mixtures with 30%, 40%, 50%, 60%, and 70% steel slag replacements increased by 8.7%, 17.3%, 22.1%, 26.9%, and 27.7%, respectively, compared with ordinary asphalt mixtures, and the growth trend gradually slowed down. When the SSP replacement amount reached 60%, the healing index slowed down significantly because the SSP dosage increased to a certain range, and the temperature during microwave healing reached the temperature required for asphalt mortar healing, therefore, the dosage cannot be increased blindly. From the comprehensive analysis of economy and healing effect, the asphalt mixture with a 60% steel slag replacement was selected as the follow-up study.

### 3.5. Analysis of Influencing Factors of Asphalt Mixture Self-Healing under Microwave Radiation

#### 3.5.1. Routine Influencing Factor Analysis

The range analysis method was used to analyze the test results of the factors affecting the self-healing of the asphalt mixture, and the asphalt mixture with 60% SSP replacement was taken as the research object, as shown in Table 9.

In Table 9, K1, K2, and K3 are the sum of indicators at each level of each factor, and $\bar{K}$1, $\bar{K}$2, and $\bar{K}$3 are the mean value of K. For example, K1 is the sum of the values of the test indicators corresponding to the “1” level, and $\bar{K}$1 is the ratio of K1 to the number of levels. R is called extreme difference, which indicates the influence range of each factor on the result, and subtracts the minimum value from the maximum value of each factor K.

**Figure 11:**
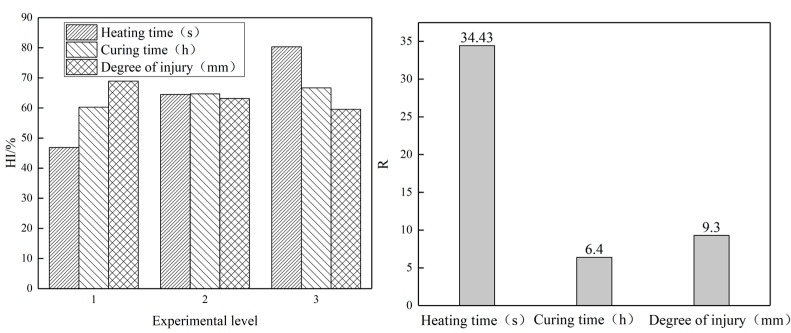
Healing index and range under different factors.

In order to visually express the experimental results, the histogram of the healing index and the range R analysis chart under different factors are shown in Figure 11.

It can be seen from Figure 11 that with the increase in microwave heating time, the increase in the average temperature of the specimen enhances the fluidity of the asphalt on the fracture surface, and the healing index HI of the specimen increases to varying degrees. The increase in curing time at room temperature was also beneficial to the healing of asphalt specimens, but the effect was small. The healing efficiency of the specimens at room temperature was low, and increasing the curing time did not significantly improve the healing effect. With the increase in the damage degree, the fracture surface gradually becomes larger, which has a negative impact on the healing of asphalt specimens. According to the range value R, the degree of influence of different factors on the healing behavior of the asphalt mixture can be obtained. The order of influence of various factors on the healing behavior of asphalt mixture is microwave heating time (healing temperature) > damage degree > curing time. Therefore, in practical application, the healing temperature should be put in the first place, followed by the timing of healing. Appropriate microwave pre-curing should be carried out when micro-cracks appear on the pavement.

#### 3.5.2. Influence of Multiple Mechanical Fatigue Damages on the Self-Healing Characteristics of Asphalt Mixture

In this section, the asphalt mixture with 60% SSP replacement and the ordinary asphalt mixture with 0% content were used as the research objects. Figure 10 shows the trend graph of the healing index along with the healing times.

**Figure 12:**
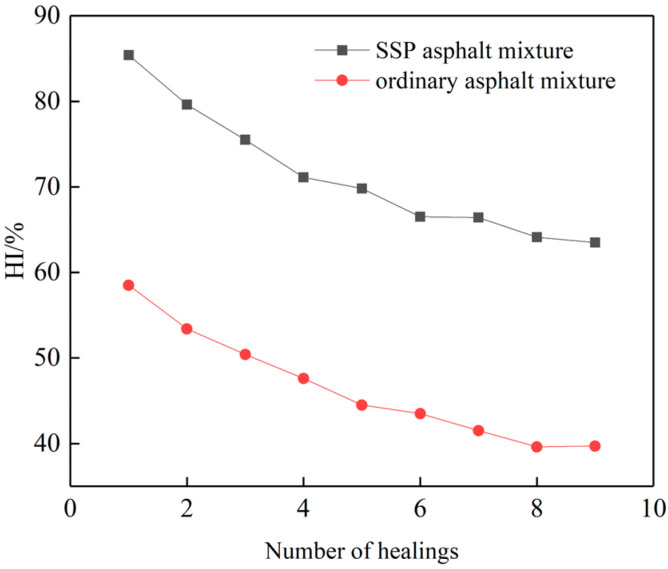
Trend chart of the healing index.

It can be seen from Figure 12 that with the increase in the number of damages, the healing index of the two asphalt mixtures generally shows a downward trend. Although the asphalt specimens can be healed many times, the performance cannot be fully recovered after each fracture. When it breaks next time, the damage will be aggravated, and the strength will gradually decrease. Compared with the ordinary asphalt mixture, the SSP asphalt mixture has a great advantage in terms of healing ability, and the healing index can still be maintained at more than 50% after repeated mechanical damage. In the actual service process of SSP asphalt pavement, the damaged pavement can be healed many times by microwave thermal-induced self-healing technology, and the service life of pavement can be extended.

## 4. Conclusions

In this paper, the feasibility of microwave-induced self-healing technology with steel slag powder was verified at two levels of binder and mixture. The following conclusions were obtained:

1. The heating rate of SSP asphalt mortar is noticeably higher than that of LP asphalt mortar under microwave radiation. There are metal oxides with high electromagnetic parameters, such as Fe_2_O_3_ and CaO in SSP, but the main component of LP is CaCO_3_. The ability of metal oxide to absorb and lose electromagnetic waves is greater than that of CaCO_3_. In addition, the surface structure of SSP is rough and porous. Electromagnetic waves will be reflected and refracted inside SSP many times, and the microwave energy consumed will be converted into heat.
2. The complex viscosity of SSP and LP asphalt mortar decreased with the increase in frequency, but when the test temperature was higher, the asphalt mortar approached or became a Newtonian fluid, and the viscosity remained unchanged with frequency. The flow behavior index n of the two asphalt mortars was proportional to the temperature but decreased with the increase in the filler–bitumen ratio. The temperature corresponding to *n* = 0.9 was selected as the most optimum healing temperature of the mortar and results showed that the optimum healing temperature of the two asphalt mortars was between 50 °C and 60 °C.
3. Under microwave radiation, the temperature distribution of the aggregate and ordinary asphalt mixture was uneven, and there was a phenomenon of temperature concentration, but the overall temperature of the asphalt mixture containing SSP increased with the increase in the SSP replacement amount, and the temperature difference decreased. From the perspective of the healing effect, the healing indexes of asphalt mixtures with 30%, 40%, 50%, 60%, and 70% steel slag replacements increased by 8.7%, 17.3%, 22.1%, 26.9%, and 27.7%, respectively, compared with ordinary asphalt mixtures, and the growth trend gradually slowed down.
4. The order of influencing factors on the microwave-induced self-healing behavior of the asphalt mixture was healing temperature > damage degree > healing time. The SSP asphalt mixture has a great advantage in terms of healing ability, and the healing index can still be maintained at more than 50% after repeated mechanical damage. In road maintenance work, the maintenance time should be controlled, and the detection of road crack damage should be carried out every year. For pavement with micro-cracks, pre-maintenance should be carried out in time to extend the service life of the pavement.

## Figures and Tables

**Table 1:** Basic properties of asphalt.

Properties	Matrix Asphalt	Requirements
Softening point (°C)	52.8	≥42
Ductility (15 °C, mm)	>1000	>1000
Penetration at (25 °C, 0.1 mm)	67.9	60–80
Density (15 °C, g/cm^3^)	1.024	–

**Table 2:** Basic properties of different fillers.

Properties	LP	SSP	Requirements
Hydrophilic coefficient	0.70	0.66	<1.00
Density (g/cm^3^)	2.68	3.39	≥2.50
Water absorption (%)	0.55	0.69	≤1.00

**Table 3:** Basic properties of aggregates.

Properties		Limestone Aggregate		
Aggregate size (mm)		0–5	5–10	10–15
Crush value (%)		/	20.6	21.2
Los Angeles wear value (%)		18.6	21.8	23.4
Apparent specific gravity (g/cm^3^)		2.724	2.723	2.737
Flat elongated particles content (%)	>9.5 mm	/	9.6	8.3
	<9.5 mm		11.4	9.2

**Table 4:** AC-13 gradation range.

Size (mm)	16	13.2	9.5	4.75	2.36	1.18	0.6	0.3	0.15	0.075
Gradation range	100	90–100	68–85	38–68	24–50	15–38	10–28	7–20	5–15	4–8
Target gradation	100	95	76.5	53	37	26.5	19	13.5	10	6

**Table 5:** Influence factors and level table.

Experiment Level	Microwave Heating Time (s)	Maintenance Time at Room Temperature (h)	Degree of Injury (mm)
1	120	2	3
2	150	3	3.5
3	180	4	4

**Table 6:** XRF test results of SSP and LP (wt%).

	CaCO_3_	SiO_2_	CaO	Fe_2_O_3_	MaO	MgO	Al_2_O_3_	Loss
SSP	-	14.2	40.5	30.5	5.2	1.9	3.2	4.5
LP	92.1	2.0	-	0.5	2.4	-	1.2	1.8

**Table 7:** The optimum healing temperature of different types of asphalt mortar.

LP Asphalt Mortar	Optimum Temperature (°C)	SSP Asphalt Mortar	Optimum Temperature (°C)
filler–bitumen ratio 0	45.5	filler–bitumen ratio 0	45.5
filler–bitumen ratio 0.6	48.4	filler–bitumen ratio 0.6	49.6
filler–bitumen ratio 0.9	53.1	filler–bitumen ratio 0.9	55.3
filler–bitumen ratio 1.2	57.2	filler–bitumen ratio 1.2	59.8

**Table 8:** Healing index of asphalt mixtures with different SSP substitutions.

Steel Slag Substitution (%)	*F*_0_ (KN)	*F*_1_ (KN)	*HI* (%)
0	9.33	5.46	58.5
30	9.52	6.39	67.2
40	9.71	7.36	75.8
50	9.82	7.91	80.6
60	10.12	8.64	85.4
70	10.76	9.28	86.2

**Table 9:** Orthogonal experimental results.

Numbering	Heating Time (min)	Maintenance Time at Room Temperature (h)	Degree of Injury (mm)	*HI* (%)
1	1	1	1	50.1
2	1	2	2	45.2
3	1	3	3	45.3
4	2	1	2	60.1
5	2	2	3	62.8
6	2	3	1	70.5
7	3	1	3	70.6
8	3	2	1	86.1
9	3	3	2	84.2
K1	140.60	180.80	206.70	
K2	193.40	194.10	189.50	
K3	240.90	200.00	178.70	
$\bar{K}$1	46.87	60.27	68.90	
$\bar{K}$2	64.47	64.70	63.17	
$\bar{K}$3	80.30	66.67	59.57	
R	33.43	6.40	9.33	

## Data Availability

Data are contained within the article.
